# Developing a Novel Web-Based Self-Management Support Intervention for Polycystic Ovary Syndrome: Mixed Methods Study With Patients and Health Care Professionals

**DOI:** 10.2196/52427

**Published:** 2024-03-07

**Authors:** Carol Percy, Andrew Turner, Charys Orr

**Affiliations:** 1 Centre for Intelligent Healthcare Coventry University Coventry United Kingdom; 2 Harris Church of England Academy Diocese of Coventry Multi Academy Trust Coventry United Kingdom

**Keywords:** anxiety, depression, PCOS, peer support, polycystic ovary syndrome, positive well-being, psychoeducation, self-management, web-based health intervention, women’s health

## Abstract

**Background:**

Polycystic ovary syndrome (PCOS) represents a significant global health burden requiring urgent attention. This common chronic endocrine and cardiometabolic condition affects around 1 in 10 women and individuals assigned female at birth, with significant adverse effects on well-being, quality of life, and mental health, as well as serious and complex long-term health consequences. International guidelines for best health care practice recommend the provision of comprehensive cognitive behavioral interventions to support self-management and improve health outcomes for those living with PCOS. Web-based health interventions have the potential to meet this need in an accessible and scalable way.

**Objective:**

We aim to identify barriers to self-management and psychological well-being in women with PCOS and adapt a web-based self-management program to provide a prototype digital support intervention for them.

**Methods:**

We adapted an existing support program (HOPE) for PCOS using the antecedent target measure approach. We conducted qualitative interviews with 13 adult women living with PCOS, 3 trustees of a patients with PCOS advocacy charity, and 4 endocrinologists to identify “antecedents” (barriers) to self-management and psychological well-being. Framework analysis was used to identify potentially modifiable antecedents to be targeted by the novel intervention. At a national conference, 58 key stakeholders (patients and health professionals) voted for the antecedents they felt were most important to address. We used research evidence and relevant theory to design a prototype for the PCOS intervention.

**Results:**

Voting identified 32 potentially modifiable antecedents, relating to knowledge, understanding, emotions, motivation, and behaviors, as priorities to be targeted in the new intervention. A modular, web-based prototype HOPE PCOS intervention was developed to address these, covering six broad topic areas (instilling HOPE for PCOS; managing the stress of PCOS; feeding your mind and body well; body image, intimacy, and close relationships; staying healthy with PCOS; and keeping PCOS in its place).

**Conclusions:**

We identified barriers to self-management and psychological well-being in women with PCOS and used these to adapt a web-based self-management program, tailoring it for PCOS, which is a comprehensive group intervention combining education, empowerment, lifestyle management, peer support with cognitive behavioral tools, and goal-setting (to be delivered by peers or codelivered with health care professionals). The modular structure offers flexibility to adapt the program further as new clinical recommendations emerge. The intervention has the potential to be delivered, evaluated for feasibility, and, if effective, integrated into health care services. Self-management interventions are not designed to replace clinical care; rather, they serve as an additional source of support. The HOPE PCOS program conveys this message in its content and activities. Future research should evaluate the prototype intervention using primary outcomes such as measures of psychological well-being, self-management self-efficacy, depression, anxiety, and PCOS-related quality of life. They should also assess the intervention’s acceptability, scalability, and cost-effectiveness.

## Introduction

### A Complex Condition With Serious Physical and Mental Health Consequences

Polycystic ovary syndrome (PCOS) represents a significant global health burden requiring urgent attention [1]. It is a common chronic cardiometabolic condition affecting around 1 in 10 women and individuals assigned female at birth. PCOS has diverse symptoms, for example, acne, alopecia, hirsutism, obesity, impaired glucose metabolism, menstrual disturbances, and subfertility [2-4]. It has significant adverse effects on well-being, quality of life, and mental health. For example, compared with healthy controls, women with PCOS have lower health-related quality of life and higher scores on symptoms of depression and anxiety [5-9], and appear to be at risk for disordered eating [10-14] and unhealthy weight management practices [15]. There is also evidence that women with PCOS may experience impaired sexual satisfaction [16], which may impact relationship satisfaction for those in intimate relationships [17]. The adverse mental health impact of PCOS may have been worsened during the COVID-19 pandemic, as patients faced an uncertain risk of contracting COVID-19, more limited access to health care services, and barriers to using their normal support strategies [18].

PCOS has serious and complex long-term health consequences [19,20]. Women with PCOS are at increased risk of sleep disturbances [21] and obstructive sleep apnea [22,23] and appear to have an elevated risk for postpartum depression [24]. They also have an increased risk of developing type 2 diabetes and cardiovascular complications [4]. Hospital admission rates for patients with PCOS have been reported to be twice as high as those without the condition [25].

### The Need for Self-Management Support

PCOS is a long-term condition with no cure, having a wide range of treatment options but no single treatment that targets every symptom or concern. Treatments are often complex and multifaceted and often include recommendations for lifestyle change, for example, altering diet and increasing physical activity [4]. Considerable self-management is required on the part of those living with PCOS, including adherence to treatment. There are wide disparities in adherence, with 1 review suggesting adherence rates ranging from 21.7% to 86% [26]. A large-scale survey suggests women with PCOS are open to adjusting their diet and physical activity to improve their health, but few achieve their goals [27].

Recent qualitative research [28-33] has identified that women with PCOS face a range of barriers to following health professionals’ lifestyle advice, including limited access to credible information, time, cost, and lack of access to safe exercise spaces, with some women lacking adequate social support, having unsupportive partners, or struggling to prioritize their own health because of responsibilities for feeding children [29]. In addition, women’s motivation and capability to adhere to recommended lifestyle changes may be impacted by the complex and multifaceted nature of PCOS, including fatigue, anxiety, depression, difficult emotions, disordered eating, the impact of stress, sleep disturbances, and a lack of critical health literacy [28,29,31-33]. Features of PCOS itself may add to the burden of following lifestyle advice, especially around weight management. For example, altered regulation of gut hormones and energy expenditure may affect women’s capability to follow diet and exercise regimes [28].

### The Need for Comprehensive, Evidence-Based, Coproduced Interventions

Patients with PCOS value specialized, integrated health care services [34], and it has been argued that the ideal model of care would be “evidence-based, patient-centered, codeveloped by consumers and health professionals” [35]. International guidelines advise (in addition to appropriate medical treatment) “Comprehensive health behavioral or cognitive behavioral interventions could be considered to increase support, engagement, retention, adherence, and maintenance of a healthy lifestyle and improve health outcomes in women with PCOS.” [4]. There is therefore scope for developing new interventions that combine education and cognitive behavioral approaches with peer support to promote physical activity, healthy eating, and emotional well-being in women with PCOS. This multifaceted approach reflects the tradition of long-term self-management that is well established in other endocrine conditions, such as diabetes [36]. Self-management is arguably broader than lifestyle change, referring to what patients must do to manage their own health in collaboration with health professionals [37,38]. This may include behavior change but also requires emotional regulation, coping, and maintaining general well-being, such as self-worth, a positive outlook, and hope [39].

### Objectives for This Study

The first aim of the study was to identify barriers to self-management and psychological well-being in women with PCOS. The second aim was to combine insights from these data to cocreatively adapt an existing web-based program to offer a prototype peer-delivered, self-management intervention to empower and support women with PCOS to enhance their psychological well-being and, if they chose to, to make appropriate lifestyle changes to self-manage their condition.

To develop our novel self-management intervention for PCOS, we selected an existing intervention that has been researched and evaluated with multiple patient groups. The HOPE program [40] is a complex intervention for long-term condition self-management with a distinctive theory and evidence base from positive psychology [41]. The HOPE program has reduced anxiety and depression and increased positive well-being for people with a range of health conditions and support needs, for example, people living with multiple sclerosis [42], parents of children with developmental disorders [43], and people living with and after cancer [44].

## Methods

### Approach to Intervention Development

The UK Medical Research Council’s (MRC) guidance on developing complex interventions [45,46] recommends that intervention development should be “a dynamic iterative process, involving stakeholders, reviewing published research evidence, drawing on existing theories, articulating program theory, undertaking primary data collection, understanding context, paying attention to future implementation in the real world, and designing and refining an intervention using iterative cycles of development with stakeholder input throughout” [46]. The most recent framework commissioned by the National Institute of Health Research and the MRC [47] further emphasizes the importance of developing interventions that are “implementable, cost-effective, transferable, and scalable in real-world conditions.” This may include adapting existing interventions and using existing, for example, digital infrastructure, rather than developing wholly new interventions ab initio. We chose to adapt and tailor an existing intervention to build on its previous successes and use its proven, scalable digital infrastructure to offer a PCOS intervention that would be as implementable, transferable, and scalable as possible.

The HOPE cancer program from which this intervention was adapted [44] was developed following the MRC recommended processes and continues to undergo refinements as each cohort or variant of the program is completed. In adapting HOPE for PCOS, we conducted an initial needs assessment with key stakeholders, reviewed published research evidence from primary sources, review papers, and international clinical guidelines, and drew on established and emerging theories about psychological well-being to produce a prototype digital intervention [48] that could be subjected to feasibility testing.

### Underpinning Theory and Evidence: Self-Management Versus Behavior Change

Interventions in the field of health behavior are more likely to be successful if they are theory-based [49]. A number of different approaches were considered for adapting the existing HOPE program, including the behavior change wheel [50] and intervention mapping [51]. However, both of these approaches require very clear specification at the outset of the intervention design of what the target behavior is that the intervention seeks to increase, decrease, promote, or modify. “Lifestyle change” is recommended as a first-line treatment in relation to the management of PCOS, and this often refers to changes to diet and physical activity. However, there is as yet no international consensus as to what precise optimal self-management behaviors might be in PCOS, and it was felt that the groundwork was lacking for the development of a behavior change intervention per se. Given the diversity of the PCOS population, for example some are lean, some are living with overweight and obesity, some are trying to conceive, others are not, some are physically active, some are more sedentary, some have issues around eating, and others are not, the development of a behavior change intervention to meet the needs of all these was beyond the scope of this study. As our objective was to adapt the existing HOPE program to support self-management, reduce depression and anxiety, and promote psychological well-being rather than develop a completely new intervention from scratch, we used the design process outlined in the following sections.

### Cocreation Process

An iterative process of cocreation was used to develop the intervention, using a mixed methodology. An initial needs assessment was conducted with key stakeholders, following the antecedent target measure (ATM) approach [52,53]. This is a flexible model that has been used in the development of previous self-management interventions, for example, for people living with cancer or dementia [54,55].

### Initial Data Collection

Semistructured interviews were conducted with women living with PCOS, trustees of the Verity UK PCOS charity who themselves had PCOS, and endocrinologists who provided services to patients with PCOS at a large publicly funded teaching hospital.

### Participant Characteristics

Those with lived experience of PCOS were 13 adult women recruited through purposive sampling from social media. All had received a formal diagnosis of PCOS from health professionals at least 6 months before the study and took part in individual interviews. The Verity trustees were 3 adult women, all diagnosed with PCOS by National Health Service (NHS) clinicians, who volunteered to be interviewed to assist with the “cocreation” of the new intervention. The health professionals interviewed in December 2016 were 4 endocrinologists working for a large UK NHS teaching hospital, which has a specialist clinic for PCOS.

### Procedure

Interviewees were presented with the problem statement “Some women with PCOS struggle to cope and self-manage effectively” and asked if they agreed and, if so, to give all the reasons why. The reasons given (“antecedents” in the terminology of ATM) were typically difficulties women experience with PCOS and in self-managing their condition. Interviews continued until participants felt they had exhausted all the antecedents they viewed as relevant for PCOS. Antecedents and the conversation through which they were generated were audio recorded and transcribed verbatim for the patient interviews. Antecedents were transcribed contemporaneously for the trustee and health professional interviews.

### Analysis

All the antecedents generated across interviews were collated and analyzed thematically following the framework analysis approach to theme development [56] to identify potential targets for intervention. When targets were chosen for intervention, only those considered to be potentially amenable to change by a self-management intervention were selected. For example, the following antecedents were excluded: endocrine characteristics of PCOS itself, social and environmental factors such as stigmatizing behavior from others, and issues with the health care system such as general practitioners’ awareness of PCOS. Once antecedents judged to be unsuitable targets for the intervention were excluded, 36 potentially modifiable antecedents remained.

### Stakeholder Voting on Priorities for Intervention

A web-based survey was created listing the 36 modifiable antecedents, and delegates at a national public engagement conference were asked to rate each of these on a 5-point scale of how important they would be to target in a self-management intervention (0=not at all important, 1=somewhat important, 2=important, 3=extremely important, and 4=crucial). Conference delegates included patients with PCOS and advocates and some health professionals treating PCOS, of whom 58 voted in the survey. All modifiable antecedents scoring 2.5 or above were retained as intervention targets; antecedents scoring below 2.5 were discarded.

### Literature Searches for Intervention Targets

Literature searches were conducted, and clinical guidelines were consulted to generate further intervention targets. Review and systematic review papers [2,16,57-68] were reviewed alongside an international clinical guideline for the management of PCOS [4]. No new targets were generated from the literature, which confirmed the findings from the needs assessment with key stakeholders.

### Adaptation of Existing Digital Intervention and Consultation With Stakeholders

The existing HOPE program has previously been specified using the Practical Reviews in Self-Management Support (PRISMS) self-management intervention taxonomy [69], and new intervention components were mapped onto or added to the program specification. New program content specific to the prototype PCOS program was created to complement the existing HOPE program materials. A paper prototype was presented to key stakeholders from the PCOS support charity, and following discussion, the new program materials were created and uploaded into the secure web-based platform that hosts the HOPE program. This produced a 6-week web-based intervention ready for feasibility testing.

### Ethical Considerations

This study received clearance from the Coventry University Research and Ethics Governance Committee (approvals P40730, P44631, and P45355). For the data collection from health care professionals, NHS Research Ethics Committee approval was not required. A letter of access for research was provided by the Research, Development, and Innovation office of the University Hospitals Coventry and Warwickshire NHS Trust. Participants provided written, informed consent before being interviewed. Patients with PCOS and health care professionals at the public engagement event completed a digital informed consent statement before completing the web-based voting survey. Interview transcripts were anonymized for analysis. No personally identifiable data were collected in the voting survey. No compensation was offered to any participants.

## Results

### Stakeholder Needs Assessment and Voting

Table 1 shows the list of modifiable antecedents presented in the next stage of the intervention design.

**Figure 1:**
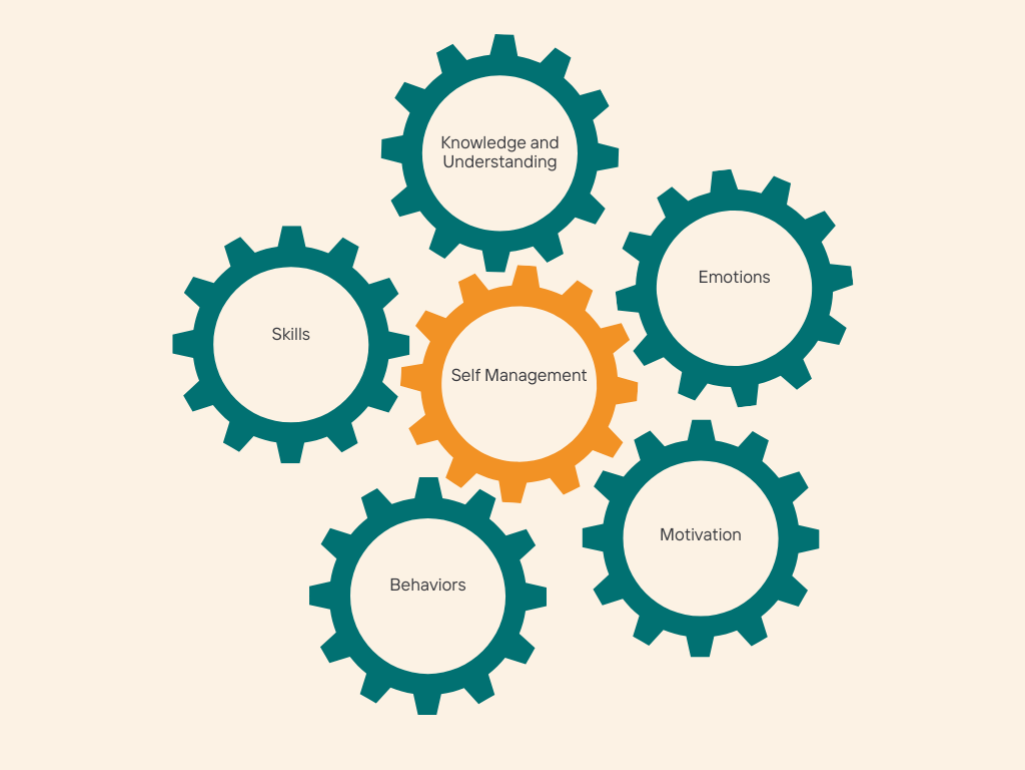
Logic model of modifiable antecedents to polycystic ovary syndrome (PCOS) self-management and psychological well-being identified in a mixed methods study of patients and health care professionals.

Figure 1 shows a diagrammatic representation of the logic model developed from the PCOS self-management needs assessment done with stakeholders.

**Table 1:** Modifiable antecedents of polycystic ovary syndrome (PCOS) self-management and psychological well-being in order of priority, as voted on by patient and health care professional delegates at the public engagement event.

Potentially modifiable antecedent	Mean score
Knowledge and understanding: long-term implications of PCOS	3.34
Knowledge and understanding: how to get the most from the health care system	3.31
Knowledge and understanding: how to eat well in PCOS	3.26
Emotions: low self esteem	3.21
Knowledge and understanding: what self-management of PCOS entails	3.19
Knowledge and understanding: how to use physical activity to manage PCOS	3.12
Knowledge and understanding: which information sources to trust	3.12
Motivation: women may find it hard to maintain motivation long-term	3.12
Emotions: feeling not properly feminine	3.09
Knowledge and understanding: the basic biology of PCOS	3.09
Emotions: depression and low mood	3.07
Behaviors: women may not follow a PCOS-healthy diet	3.02
Emotions: struggling to make healthy choices	3.02
Behaviors: women may eat in disordered ways, for example, binge eating and overeating	3.00
Motivation: women may lack motivation to self-manage	3.00
Emotions: embarrassment and shame	2.95
Motivation: women may have low confidence to self-manage	2.95
Behaviors: women may not use physical activity to manage their PCOS effectively	2.91
Emotions: lack of trust in health professionals	2.91
Emotions: anxiety and fear	2.90
Behaviors: women may not prioritize their own needs effectively	2.88
Motivation: women may lack clear self-management goals	2.79
Behaviors: women may avoid or give up on self-management	2.76
Emotions: frustration and anger	2.71
Emotions: loneliness and isolation	2.71
Motivation: women may not find physical activity enjoyable	2.67
Skills: women need better skills to communicate needs to health professionals	2.62
Behaviors: women may not self-monitor their menstrual cycle effectively	2.60
Motivation: women may be in denial and avoid trying to self-manage	2.60
Motivation: women may have unrealistic goals	2.59
Knowledge and understanding: women may have unrealistic expectations of the female body	2.57
Behaviors: women may isolate themselves from social contact	2.52
Behaviors: women may hide their self-management activity from others^a^	2.47
Motivation: women may not be ready yet to start self-managing^a^	2.41
Behaviors: women may not take medications as prescribed^a^	2.21
Behaviors: women may self-harm^a^	2.17

^a^Potential antecedents scoring below 2.5 were discarded as intervention priorities. This left 32 antecedents that were targeted by the new intervention, 10 of which were thematically categorized in the framework analysis under behaviors, 9 under emotions, 8 under knowledge and understanding, 8 under motivation, and 1 under skills.

In a typical ATM design, logic models are often presented as linear processes in which 1 antecedent, such as knowledge or understanding, is represented as preceding another, such as motivation or behavior. We have selected the metaphor of interlocking gears for our schematic logic model on the assumption that knowledge, understanding, skills, motivation, emotions, and behavior interlock and may drive or impede each other, at times facilitating or hampering effective self-management. For example, a person might gain helpful knowledge about their condition, but without developing skills to apply this to practice, they might be unable to engage in effective self-management behaviors. Similarly, a person might have adequate knowledge, understanding, and skills to manage their condition, but difficult emotions might adversely impact their motivation and prevent effective self-management. This logic model was used to inform the adaptation of the existing HOPE program framework to produce a specific version for PCOS.

### Mapping Modifiable Antecedents Onto Intervention Content

Table 2 shows how the antecedent “problems” derived from the logic model were mapped onto solution-focused content across 6 sessions of the prototype intervention.

**Table 2:** Mapping of antecedents of polycystic ovary syndrome (PCOS) self-management and psychological well-being onto new PCOS self-management intervention session content.

Potentially modifiable antecedent	Course sessions					
	1	2	3	4	5	6
Long-term implications of PCOS	✓		✓		✓	
Getting the most from the health care system	✓			✓	✓	✓
How to eat well in PCOS		✓	✓			
Coping with low self esteem	✓	✓	✓	✓	✓	
What self-management of PCOS entails	✓	✓	✓	✓	✓	
How to use physical activity to manage PCOS	✓	✓	✓			
Which information sources to trust	✓	✓	✓	✓	✓	✓
Maintaining motivation longer term	✓	✓	✓	✓	✓	✓
Coping with feeling “not properly feminine”	✓		✓	✓		✓
Basic biology of PCOS	✓		✓	✓	✓	✓
Coping with depression and low mood	✓	✓	✓	✓	✓	✓
Following a PCOS-healthy diet	✓		✓			
Making healthy choices	✓		✓			
Coping with eating distress, for example, binge eating and overeating			✓			
Motivation to self-manage	✓	✓	✓	✓	✓	✓
Coping with embarrassment and shame	✓	✓	✓	✓		✓
Confidence to self-manage	✓	✓	✓	✓	✓	✓
Using physical activity to manage PCOS	✓	✓	✓			
Trust in health professionals	✓		✓	✓	✓	✓
Coping with anxiety and fear	✓	✓	✓	✓	✓	✓
Prioritizing your own needs	✓	✓	✓	✓	✓	✓
Setting clear self-management goals	✓	✓	✓	✓	✓	✓
Not giving up on self-management	✓	✓	✓	✓	✓	✓
Coping with frustration and anger	✓	✓	✓	✓	✓	✓
Coping with loneliness and isolation	✓	✓	✓	✓	✓	✓
Finding a physical activity that is enjoyable		✓				
Skills to communicate with health professionals	✓			✓	✓	
Monitoring your menstrual cycle	✓	✓		✓		✓
How to keep trying to self-manage	✓	✓	✓	✓	✓	✓
Setting realistic goals	✓	✓	✓	✓	✓	✓
Realistic expectations of the body	✓			✓		
Getting social support	✓	✓	✓	✓	✓	✓

### Choosing Theory- and Evidence-Based Intervention Components to Address Modifiable Antecedents of Self-Management

The HOPE program framework used to develop the PCOS intervention includes the following self-management intervention components: information about available resources, safety netting, training and rehearsal to communicate with health care professionals, training and rehearsal in psychological strategies, social support, and lifestyle advice and support [69]. Below, we outline how specific intervention content was selected to address the antecedents identified in the logic model for PCOS self-management.

### Information and Activities to Target Knowledge and Understanding for Self-Management

Women with PCOS have unmet information needs [63,70-72] and may lack knowledge of how to manage their condition, for example, how to eat well to manage insulin resistance [33,73]. Unmet information needs may adversely impact self-management, including adherence to treatment [26,28,32,74,75]. PCOS-specific information is valued by patients with PCOS and may address unmet psychosocial needs [76-78], potentially enhancing understanding, reducing anxiety, and promoting quality of life [79]. There is a plethora of information sources available on the web, of varying quality, making it difficult for women with PCOS to select trustworthy material [71,80-83]. Signposting to trustworthy information sources may support patient activation and self-management. In the prototype intervention, factual information is provided about PCOS, including androgen excess and food intake, blood sugar and insulin resistance, eating well, and managing weight.

The needs assessment and previous research [84,85] indicate women may have concerns about the long-term health sequelae of PCOS. Motivation to adopt healthy lifestyles may be impacted by perceptions of the health risks associated with PCOS [28]. In the prototype intervention, content is provided on focusing on future health and ways to manage emerging health concerns and worries. Information about specialist health professionals, the range of support, and some potential treatment options is designed to empower participants to be active partners in their health care. The intervention seeks to empower participants to engage in health protective behaviors, for example, exercise, healthy eating, self-monitoring of their health, managing worries to alleviate health anxiety, and future help-seeking as any new health issues arise. Links to further information and resources are provided, for example, Verity (a UK PCOS charity), the NHS Choices website, the NHS Improving Access to Psychological Therapies service, and the AskPCOS app [86].

### Goal Setting and Feedback to Target Self-Management Motivation and Behavior

Women with PCOS may struggle to achieve the goals they set for lifestyle change [27], may lack the ability to identify and resolve barriers [32], experience tiredness, feel unrewarded, or have depressive and defeating thoughts that act as barriers to achieving their goals [29]. Goal setting is a widely used and effective component of self-management support [87,88]. Goal-oriented care, emphasizing patient priorities and values, may be particularly appropriate for patients with chronic or multifaceted health concerns [89]. Goal-setting theory and research recommend paying attention to multiple goal factors and multiple phases in the goal process [90].

Goal setting and solution-focused goal feedback are included in every session of the prototype intervention, with weekly and long-term goals chosen by participants themselves based on their personal priorities and values rather than set or recommended by program facilitators. The goal-setting and feedback processes are facilitated by peer facilitators, with attention to goal difficulty, goal specificity, goal proximity, goal commitment, and solution-focused feedback.

### Psychoeducation and Activities to Target Emotions

Women with PCOS are prone to higher perceived stress [91], and stress may also impact women with PCOS differently and have an impact on the physiology of the condition [92,93]. Stress may be a barrier to lifestyle change [32]. Mindfulness-based stress reduction activities have been effective in reducing stress, depressive, and anxiety symptoms in PCOS [94] and in increasing self-efficacy for physical activity and nutrition behaviors [95]. Physical activity has well-recognized benefits for mood and perceived stress [96,97], including in women with PCOS [98,99]. In the prototype intervention, content is provided on managing stress, including mindfulness, soothing rhythm breathing, guided imagery, and “get active, feel good.” Because interviews with health professionals during the needs assessment suggested that some women with PCOS may exercise in dysfunctional ways in order to lose weight, the PCOS prototype intervention does not promote exercise for weight loss per se but rather for stress management and general well-being.

Psychological comorbidities may be a barrier to lifestyle change [32]. Interventions using aspects of cognitive behavioral therapy (CBT) have shown promise for improving fatigue, quality of life, weight loss, and depressive symptoms in women with PCOS [100-103]. In the prototype intervention, CBT-informed content is provided on managing common unhelpful thinking patterns. Compassionate mind approaches (sometimes referred to as part of a “third wave” of CBT) are particularly relevant to disordered eating, emotional eating, and weight management [104-107]. In the prototype intervention, a compassionate mind approach is taken to eating well, including eating mindfully, overeating, binge eating, and self-soothing.

Self-compassion activities, compassionate mind training, and compassion-focused therapy have been demonstrated to be particularly beneficial in improving anxiety, depression, shame, and self-criticism [106,108-111]. These issues have been identified as common in populations with PCOS. In the prototype intervention, content is provided on self-compassion, including, for example, compassion for perceived flaws, toward feelings of embarrassment or shame, and “getting to know your inner critic.”

Women with PCOS may have reduced relational and marital satisfaction [17,112], sexual satisfaction, function, and sense of sexual attractiveness [16,113-119]. Body dissatisfaction may be a barrier to lifestyle change [28]. The prototype intervention discusses and seeks to normalize the topic of common body changes and associated distress, for example, acne, alopecia, hirsutism, obesity, and associated low self-esteem, frustration, and depression, which have been identified in previous research [84,120-123], and in the needs assessment. Content is provided on common body changes in PCOS and associated difficult emotions. The potential impact of PCOS on intimate relationships and sexuality is explored, along with suggestions for ways to cope and adjust.

The population with PCOS is diverse, including women who will be more or less concerned about visible or invisible body changes. Interventions may empower them to respond and adjust in ways that are personally appropriate, including accepting changes, making adaptations, or seeking treatment, for example, for obesity or hirsutism. Previous research, including the needs assessment, indicates that embarrassment or shame may lead women to conceal the extent of their bodily changes, even from health professionals [124-127]. In the prototype intervention, content is provided on responding to change, including acceptance, treatments and adaptations, and overcoming embarrassment to get help for body changes. The PCOS intervention takes an autonomy or body acceptance approach [128,129]. Unrealistic female body standards and body-positive activism are discussed in the program materials.

Women may struggle to manage and control their PCOS symptoms, especially if they have comorbidities, sometimes feeling controlled by their condition [84]. Motivational imagery interventions are widely used in sports psychology, with benefits for motor performance, motivational outcomes, and affective outcomes [130] and found to be motivating by a number of different populations, including people trying to manage their weight or type 2 diabetes [131,132]. Emotional mental imagery interventions have shown promise for reducing anxiety and depression [133,134]. Prospective (future-focused) mental imagery interventions are particularly relevant to fostering optimism in people with depression [135]. In the prototype intervention, content is provided on life priorities and motivational imagery—“keeping PCOS in its place”—and future-focused mental imagery.

Positive psychology interventions, such as character strengths and gratitude diaries, which focus on function rather than dysfunction, have shown promise in treating anxiety, depression, low mood, and low self-esteem and in promoting positive well-being [136-138]. In the prototype intervention, content is provided on maximizing psychological resources, for example, by focusing on character strengths and gratitude diaries rather than focusing purely on psychological deficits and dysfunctions.

### Activities for Development of Self-Management Communication Skills

Being able to communicate the need for practical and social support is essential for effective self-management. Communicating with friends, family, peers, and health professionals may be particularly difficult for women with PCOS because of its sometimes visible, sometimes invisible nature and associated taboos and stigma [139-142]. In the prototype intervention, content is provided on communication, including communicating concerns with friends, family, peers, and health professionals.

The intervention explicitly acknowledges the issue that some women with PCOS may lack trust in, or feel dismissed or stigmatized by, health professionals [70,71,143-147]. Some health professionals treating PCOS recognize that “lifestyle change,” especially around weight, is a sensitive topic [148], but some patients report a lack of trust in and perceived lack of empathy from health professionals. Intervention materials in the prototype explore and deconstruct health communication around weight to foster shared understanding and trust.

People with long-term health conditions need a range of knowledge and skills to navigate health care systems and participate actively in consultations and care [149]. In the prototype intervention, content is provided on maximizing support from health services and health professionals, including health care specialists who treat PCOS, treatments, “why it may sometimes sound like health professionals are being preachy or judgemental,” summarizing concerns and requesting a referral, shared agenda setting at health care appointments, and communicating clearly and assertively with health professionals.

### Building on Therapeutic Peer Group Factors and Collective Advocacy

Women with PCOS are interested in group education interventions [150]. Group psychosocial and psychoeducational interventions provide particular therapeutic factors, for example, instillation of hope, universality, imparting of information, and opportunities for altruism and catharsis [151]. A lack of social support may be a barrier to lifestyle change [32,33]. Group and peer support, provided face-to-face or on the web, have been beneficial for those living with PCOS [76,78,152-154]. Peers delivering interventions may be well placed to express empathy and to act as realistic role models, supporting self-management [155-157].

Peer group factors are emphasized and facilitated throughout the prototype PCOS intervention, including, for example, self-management, “your PCOS journey,” getting peer support, open space forums, support from health professionals, peers, and the UK PCOS charity, and signposting to ongoing participation with PCOS-related groups and networks. The intervention is delivered in a web-based group format with secure digital social sharing features, for example, a gratitude wall, hopes and dreams, goal sharing, and delivery by trained peer facilitators who themselves had PCOS. Group delivery allows the intervention to be made available to multiple participants, saving time and resources for facilitators.

Improving health care access and outcomes for PCOS is not simply a matter of individual patient assertiveness. Collective collaborative action between patients, health professionals, and powerful others is needed to bring about change. In the prototype intervention, content acknowledges the limits of personal assertiveness and emphasizes the value of collective action. For example, we signpost to activism by Verity [158] and other women’s health organizations.

### Modular Web-Based Format for Accessibility And Scalability

Digital health interventions offer considerable scope for accessibility and scalability [159]. The prototype PCOS intervention is designed to be delivered digitally through a secure social platform developed by the social enterprise community interest company Hope for the Community (H4C) [160]. The social enterprise company hosts the intervention and has a track record of scaling up the existing HOPE interventions for a range of commissioners. The H4C web-based intervention platform allows peers to support each other and engage in synchronous or asynchronous therapeutic activities in a web-based space without the logistical and practical difficulties of travel and finding venues and times suitable for all.

### Program Specification

Textbox 1 shows the content of the 6-week prototype intervention program. Solution-focused goal setting and feedback, open forum discussions, and further resources are provided in every session.

The H4C team uploaded the additional content and created a working prototype intervention that was checked and tested, ready for a pilot study with women living with PCOS.

**Content of polycystic ovary syndrome (PCOS) intervention.**

**Session 1: Instilling HOPE for PCOS**

- Welcome and introductions
- Responsibilities and ground rules
- Instilling HOPE
- Diaphragmatic breathing
- Gratitude diary
- Your PCOS journey
- Support from health professionals, peers, and Verity (UK PCOS charity)
- PCOS basics: androgen excess & insulin resistance
- Test your PCOS basics: quiz
- Factual information about PCOS
- Self-management
- Self-compassion

**Session 2: Managing the stress of PCOS**

- Gratitude diary
- Managing stress
- Mindfulness
- Physical activity for stress management: “get active, feel good”
- Managing common unhelpful thinking patterns (cognitive behavioral therapy [CBT])
- Mindfulness: and am I doing this “right”?
- Why self-compassionate mindfulness?
- Compassion-focused therapy

**Session 3: Feeding your mind and body well**

- Gratitude diary
- Eating well in PCOS: role of food intake, blood sugar, and insulin
- Why being insulin resistant is a problem for the whole body
- Why it may sometimes sound like health professionals are being preachy or judgmental
- Eating well without depriving yourself, losing weight in PCOS
- Eating mindfully to eat well in PCOS
- Overeating and binge eating in PCOS: some helpful tips for reducing the chances of overeating
- Feeding your mind and body well
- Feeding your mind: three systems regulating our emotions
- Ways to soothe yourself without over- or undereating
- Getting to know your inner critic, developing a compassionate ideal

**Session 4: Body image, intimacy, and close relationships**

- Gratitude diary
- Body changes, sexuality, and intimacy
- Communication
- Common body changes in PCOS
- Difficult emotions that can come with body changes
- Getting ready to be self-compassionate
- Compassion for your perceived flaws
- Overcoming embarrassment to get help for body changes
- Getting ready to be self-compassionate toward feelings of embarrassment or shame
- Responding to change: acceptance, treatments, and adaptations
- Treatments

**Session 5: Staying healthy with PCOS**

- Gratitude diary
- Focusing on your future health
- Ways to manage emerging health concerns and worries about your future health
- Getting peer support
- Maximizing your psychological resources
- Maximize the support you get from health services and health professionals
- Health care specialists who treat PCOS
- Activity: Summarizing concerns and requesting a referral
- Communicating clearly and assertively with health professionals
- Activity: Shared agenda setting at health care appointments
- When communicating clearly and asking assertively do not get you what you want

**Session 6: Keeping PCOS in its place—your strengths and life goals**

- Gratitude diary
- Character strengths
- Life priorities
- Motivational imagery
- Sharing our successes
- Self-compassion
- Activity: self-compassionate letter
- Signposting to ongoing participation with PCOS related groups and networks

## Discussion

### Principal Results

We cocreatively identified and prioritized barriers to self-management and psychological well-being to adapt a successful web-based self-management program and tailor it for the needs of adult women with PCOS. We developed a web-based prototype program for the PCOS program, ready for testing with this population.

### Comparison With Previous Work

Women with PCOS value specialized, integrated, evidence-based, patient-centered health care services, ideally codeveloped with consumers and health professionals [34,35]. The new HOPE PCOS intervention has been codeveloped by combining input from patients, patients with PCOS’ advocates, and health care professionals. It is a comprehensive intervention integrating education and empowerment with lifestyle management, cognitive behavioral tools, and peer support, ready to be delivered by trained peer facilitators or codelivered by peers and health care professionals.

Developed following MRC guidance for the development of complex interventions [45,47], the intervention is evidence-based, underpinned by relevant theory, and designed to support a collaborative approach to care. Subject to evaluation, the new intervention could be integrated as part of PCOS services to support self-management and the model of collaborative, patient-centered health care advocated by international guidelines [4,161].

The intervention deploys an existing secure web-based social platform that has been used successfully to scale up other self-management support programs for other populations and conditions [40,44]. The multicomponent and modular structure of the program offers flexibility in adapting it further as new clinical recommendations and patient information emerge. The intervention is therefore well placed to be delivered and evaluated at scale.

The HOPE PCOS intervention is designed to support self-management and promote psychological well-being, rather than as a behavior change intervention per se. Since we started the process of cocreating the HOPE PCOS intervention, a different team has undertaken development work for a behavior change lifestyle intervention using a different intervention design model [28,31-33]. That work has not yet, to our knowledge, resulted in a prototype intervention. However, the findings from the needs assessment work conducted by that team for their lifestyle intervention concur considerably with our own needs assessment results, showing that women living with PCOS may often experience barriers of capability, opportunity, and motivation, for example, a lack of credible information, difficulty managing multiple health conditions, limited access to resources, adequate social support, and issues such as health expectancies and emotional eating affecting their motivation to engage in lifestyle change [33].

### Limitations

The current HOPE PCOS intervention is based on a needs assessment conducted with adult women. As part of the iterative cocreation process, further needs assessments may be required to adapt and tailor future versions of the intervention for other populations, for example, adolescents with PCOS, as concerns, distress, and intervention format preferences may differ [162]. There is scope to develop variants of the intervention for specific adult populations, for example, perimenopausal, menopausal, or postmenopausal women with PCOS who may have specific concerns [163-165]. The intervention might also be tailored to support trans and nonbinary people with PCOS, those with particular concerns such as weight management, comorbid conditions, or those actively trying to conceive. More cocreation and development work could be done to ensure the intervention is culturally appropriate. The intervention is intended to provide extra support and not to bypass what is recommended by a clinician. Women with PCOS are not advised to rely fully on this self-management intervention and are advised to consult a clinician as appropriate.

Cocreative intervention development is an iterative process, and, in addition to tailoring the HOPE PCOS intervention for different populations, future iterations may need to develop new content or place greater emphasis on some components, depending on how participants and health professionals evaluate the program. If consensus emerges about specific target behaviors for self-management in PCOS, it may be necessary in the future to adapt the intervention to promote specific behavior change. For example, the current content promoting physical activity could be modified and refined to promote a target level of physical activity, or the current material on healthy eating could be modified to promote specific changes in eating behavior.

Although the prototype intervention has been collaboratively co-designed to support self-management and psychological well-being, further feasibility research is needed with patients with PCOS using the program. This should evaluate the program’s acceptability as well as its impact on key outcomes such as measures of self-management, depression, anxiety, and psychological well-being. Future work is also needed to assess the intervention’s scalability, cost-effectiveness, and suitability for integration with standard care.

### Conclusions

The development of the novel HOPE PCOS intervention contributes to ongoing efforts to support patients with long-term conditions to self-manage effectively and is, to our knowledge, the first such program for patients with PCOS. This study has demonstrated that comprehensive intervention programs can be codeveloped with patients with PCOS, patient advocates, and health care professionals to address multiple barriers to self-management and psychological well-being. A person-centered, holistic approach, organized around the self-reported needs and priorities of patients, may produce interventions that complement services provided by health care professionals and patient advocacy and support organizations.

This study demonstrates that the use of a remote digital platform with preloaded, evidence-based intervention content may offer economies of scale in that complex self-management support interventions might be delivered to many patients in parallel, potentially saving clinic resources for one-to-one care. A key message for those working to develop lifestyle or self-management support interventions is the potential value of adapting an existing program and reusing existing digital infrastructure to provide a tailored intervention without the additional resource implications of developing an entire intervention from scratch.

## Abbreviations

ATM: antecedent target measure
CBT: cognitive behavioral therapy
H4C: Hope for the Community
MRC: Medical Research Council
NHS: National Health Service
PCOS: polycystic ovary syndrome
PRISMS: Practical Reviews in Self-Management Support

## Data Availability

The data sets generated or analyzed during this study are not publicly available due to participants’ consent being given for anonymized extracts only (not whole transcripts) to be used in published reports, but data are available from the corresponding author upon reasonable request.
